# Detection of Alzheimer Disease in Neuroimages Using Vision Transformers: Systematic Review and Meta-Analysis

**DOI:** 10.2196/62647

**Published:** 2025-02-05

**Authors:** Vivens Mubonanyikuzo, Hongjie Yan, Temitope Emmanuel Komolafe, Liang Zhou, Tao Wu, Nizhuan Wang

**Affiliations:** 1 College of Health Science and Engineering University of Shanghai for Science and Technology Shanghai China; 2 Department of Neurology Affiliated Lianyungang Hospital of Xuzhou Medical University Lianyungang China; 3 Collaborative Research Center Shanghai University of Medicine & Health Sciences Shanghai China; 4 Department of Radiology Jiading District Central Hospital Affiliated to Shanghai University of Medicine & Health Sciences Shanghai China; 5 Department of Chinese and Bilingual Studies The Hong Kong Polytechnic University Kowloon China (Hong Kong)

**Keywords:** diagnostic accuracy, vision transformer, Alzheimer disease, detection, neuroimaging, meta-analysis, neuroimaging, deep learning, medical database, diagnostic, clinical implementation, machine learning, magnetic resonance imaging, neural networks

## Abstract

**Background:**

Alzheimer disease (AD) is a progressive condition characterized by cognitive decline and memory loss. Vision transformers (ViTs) are emerging as promising deep learning models in medical imaging, with potential applications in the detection and diagnosis of AD.

**Objective:**

This review systematically examines recent studies on the application of ViTs in detecting AD, evaluating the diagnostic accuracy and impact of network architecture on model performance.

**Methods:**

We conducted a systematic search across major medical databases, including China National Knowledge Infrastructure, CENTRAL (Cochrane Central Register of Controlled Trials), ScienceDirect, PubMed, Web of Science, and Scopus, covering publications from January 1, 2020, to March 1, 2024. A manual search was also performed to include relevant gray literature. The included papers used ViT models for AD detection versus healthy controls based on neuroimaging data, and the included studies used magnetic resonance imaging and positron emission tomography. Pooled diagnostic accuracy estimates, including sensitivity, specificity, likelihood ratios, and diagnostic odds ratios, were derived using random-effects models. Subgroup analyses comparing the diagnostic performance of different ViT network architectures were performed.

**Results:**

The meta-analysis, encompassing 11 studies with 95% CIs and *P* values, demonstrated pooled diagnostic accuracy: sensitivity 0.925 (95% CI 0.892-0.959; *P*<.01), specificity 0.957 (95% CI 0.932-0.981; *P*<.01), positive likelihood ratio 21.84 (95% CI 12.26-38.91; *P*<.01), and negative likelihood ratio 0.08 (95% CI 0.05-0.14; *P*<.01). The area under the curve was notably high at 0.924. The findings highlight the potential of ViTs as effective tools for early and accurate AD diagnosis, offering insights for future neuroimaging-based diagnostic approaches.

**Conclusions:**

This systematic review provides valuable evidence for the utility of ViT models in distinguishing patients with AD from healthy controls, thereby contributing to advancements in neuroimaging-based diagnostic methodologies.

**Trial Registration:**

PROSPERO CRD42024584347; https://www.crd.york.ac.uk/prospero/display_record.php?RecordID=584347

## Introduction

Alzheimer disease (AD) is a progressive neurodegenerative disorder characterized by deterioration in cognitive function and memory impairment. As the global population ages, AD is emerging as a significant public health concern, affecting millions of individuals worldwide [1]. AD poses a mounting socioeconomic burden due to the escalating costs associated with medical care and long-term support for patients with these diseases and their caregivers. Consequently, there is an urgent need to develop accurate and early diagnostic methods to facilitate timely intervention and management of this debilitating condition [2].

The early and accurate detection of AD is imperative for initiating timely therapeutic interventions and proper care planning before irreversible neurological damage occurs. However, a definitive diagnosis of AD can only be established postmortem through the identification of amyloid plaques and neurofibrillary tangles in the brain tissue. Consequently, researchers have turned their attention to leveraging novel imaging techniques, such as magnetic resonance imaging (MRI) and 18-fluorodeoxyglucose and positron emission tomography, by the use of advanced computational techniques like machine learning (ML) and deep learning (DL) approaches to facilitate the earlier identification of AD. These advanced neuroimaging modalities and computational methods hold the potential to aid in the presymptomatic detection and monitoring of AD progression, which enables more effective disease management strategies [3]. DL and ML approaches have been widely applied to various diagnostic tasks, although the diagnostic accuracy of DL remains uncertain in some areas. To address this gap, meta-analysis methods have been proposed [4] to provide a more comprehensive understanding. For example, studies have estimated the diagnostic accuracy of DL models for COVID-19 detection [5] and evaluated ML models for osteoporosis diagnosis in the hip bone [6]. Similarly, both ML and DL techniques have been extensively used for the detection and diagnostic evaluation of AD. Odusami et al [7] undertook a systematic review and meta-analysis. They used ML models alongside multimodal neuroimaging data to classify various stages of AD progression. The findings from their investigation were highly encouraging, demonstrating pooled estimates for sensitivity of 94.6% and specificity of 93.5% in classifying patients with AD from healthy controls. This study demonstrates the considerable promise of ML algorithms when combined with multimodal neuroimaging biomarkers for differentiating patients with AD from cognitively normal (CN) individuals. This capability holds potential advantages for enabling early diagnosis and disease monitoring, essential for effective management and treatment. The application of ML in AD diagnosis opens a new chapter for research with the capacity to significantly impact patient care and management. The extensive meta-analysis conducted by Wang et al [8] explored the concept of deep neural networks and heat map visualizations on multimodal neuroimaging data, encompassing structural MRI and functional imaging, to unravel patterns linked with AD. Through the application of deep neural networks and heat map visualizations, the study successfully pinpointed and emphasized the particular brain regions mostly impacted by the disease. This outcome provides valuable insights into the fundamental mechanisms underlying AD. Qu et al [9] conducted a comprehensive review and meta-analysis comparing the diagnostic efficacy of generative adversarial network (GAN)–based and non-GAN DL approaches in AD diagnosis. Their findings revealed that GAN-based DL models are more superior and effective than the non-GAN models in distinguishing individuals with AD from those with just CN individuals. This superiority was demonstrated through significant enhancements in accuracy, sensitivity, specificity, and the area under the curve of the summary receiver operating characteristic. However, when differentiating between progressive mild cognitive impairment (MCI) and stable MCI, the GAN method did not demonstrate a notable improvement in either the accuracy or the sensitivity. Despite this, it displayed marginally better specificity and area under the curve of the summary receiver operating characteristic curve compared with the non-GAN method. These results highlight the potential of GAN-based DL techniques to enhance AD diagnosis. Nonetheless, their applicability may be constrained in certain diagnostic contexts. Vision transformers (ViTs) are a novel type of DL model that has been effectively used in various types of computer vision tasks due to their ability to learn long-range connections within the images [10]. The ViTs process image data using the self-attention mechanism, which allows the model to connect sensitive regions of the image, which ultimately improves computing efficiency and contextual comprehension. Dosovitskiy et al [11] proposed a novel approach in computer vision, demonstrating the efficacy of ViT for image recognition tasks. Deviating from conventional methods that rely on convolutional neural networks (CNN), ViTs directly apply transformer architecture to sequences of image patches. By leveraging pretraining on extensive data sets, ViTs achieve competitive performance on various image classification benchmarks with lesser computational complexity. This pioneering study emphasizes the potential of transformer-based models in revolutionizing image recognition tasks, with scalable and promising results. Thereafter, various novel ViT models were developed, with enhancements aimed at reducing their computational cost and extending them to additional vision tasks, such as object detection [12] and image segmentation [13]. ViTs are well-designed to serve as the fundamental models for computer vision tasks in the medical field. Therefore, many studies have been published recently focusing on this area of research [14-17].

CNN models process images as pixel arrays, whereas ViT splits the input images into visual tokens. The visual transformer breaks an image into fixed-size patches, embeds each one appropriately, and incorporates positional embeddings as input for the transformer encoder. In addition, ViT models offer nearly four times the computational efficiency and accuracy compared with CNNs [18]. The self-attention layer in ViT enables the model to embed information globally across the entire image. It also learns from the training data to encode the relative positions of image patches, allowing it to reconstruct the image’s structure effectively.

Despite the promising outcomes documented in the scientific literature regarding the utilization of ViTs for AD image classification and analysis, a comprehensive meta-analysis examining the advantages and performance of these ViTs is notably absent. This research gap underscores the necessity for a systematic review and meta-analysis to consolidate existing evidence systematically; this systematic review with meta-analysis of ViT network architecture aims to address this evidence gap. The goal of this review and meta-analysis is to evaluate and summarize all available evidence to quantitatively assess the diagnostic test accuracy (DTA) of ViTs for detecting AD using MRI and positron emission tomography (PET) brain images, also providing critical insights that can inform future research directions and clinical practice in the field of AD diagnosis and management.

## Methods

### Data Sources and Literature Query

In this systematic review and meta-analysis, relevant studies on AD and ViT-based models were systematically gathered from multiple databases known for extensive research on these topics. These databases included CENTRAL (Cochrane Central Register of Controlled Trials), China National Knowledge Infrastructure, PubMed, Scopus, ScienceDirect, and Web of Science. In addition, we searched other sources like Google Scholar for gray literature and conducted citation searches. In total, 24,693 records were identified. The selected literature encompassed publications from January 1, 2020, to March 1, 2024, specifically focusing on studies using ViTs for AD prediction with neuroimaging data. The selected studies used MRI and PET as imaging data. The search strategy used MeSH terms and other selected keywords across different databases. Search strings can be found in Multimedia Appendix 1. A comprehensive outline of the search methodology, adhering to the Preferred Reporting Items for Systematic reviews and Meta-Analyses (PRISMA) guideline of reporting systematic reviews [19], can be found in Multimedia Appendix 2.

### Eligibility Criteria

In this systematic review and meta-analysis, we considered studies using ViTs for AD image classification, specifically those published since their inception in 2020 [11]. These studies were required to report detailed performance metrics, including sensitivity, specificity, and a 2×2 confusion matrix, for ViT models used either independently or in hybrid models with other DL architectures. The MRI and PET image data sets used in the studies could be either unimodal or multimodal. Studies were excluded based on the following criteria: (1) conference abstracts, (2) duplicate studies, (3) incomplete papers lacking full text, (4) studies lacking classification results for AD and normal control (NC), (5) studies not using ViT models for AD detection, (6) literature reviews, and (7) studies not adopting neuroimaging for AD and NC prediction. Studies included in this review showcased the application of ViT models for AD image classification and provided comprehensive performance metrics.

### Selection of Involved Studies

Two teams of reviewers (VM and TEK and HY and NW) independently conducted the screening process. Using Rayyan (Rayyan Systems Inc) [20], a freely available software, duplicates were removed based on titles or abstracts, streamlining the screening process. The initial review involved titles and abstracts, followed by a thorough examination of full texts for further screening. Eligible studies were included in the final analysis. In instances where the 2 review teams were unable to reach an agreement, the disagreement was resolved by consulting a more experienced third reviewer (LZ), who facilitated consensus.

### Data Collection Process

Two researchers (VM and TEK) independently adopted a self-developed data extraction form to meticulously extract specific parameters regarding network architecture. These include the country of study, network model type, data type, methods, number of training epochs, batch sizes, and other relevant information. In addition, we diligently extracted the (2×2) confusion matrix, consisting of true positive, false negative, true negative, and false positive. These data formed the foundation for computing various DTA metrics, including sensitivity, specificity, diagnostic odds ratios (DORs), recall, precision, *F*_1_-score, and positive and negative likelihood ratios (LR+ and LR–). This rigorous approach ensures a comprehensive analysis of ViT performance in AD detection using neuroimages. These metrics were used to assess the performance of models used in studies. The formulas for estimating some of these parameters are presented in Equations 1-4.



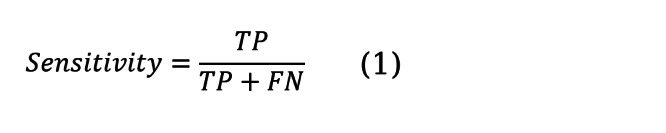





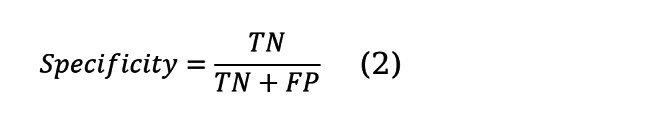





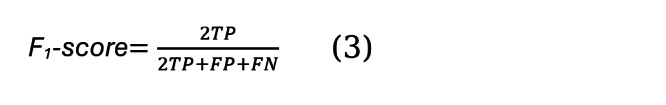





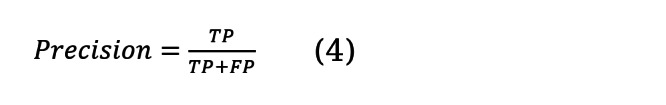



### Risk of Bias and Quality Assessment

**Figure 2:**
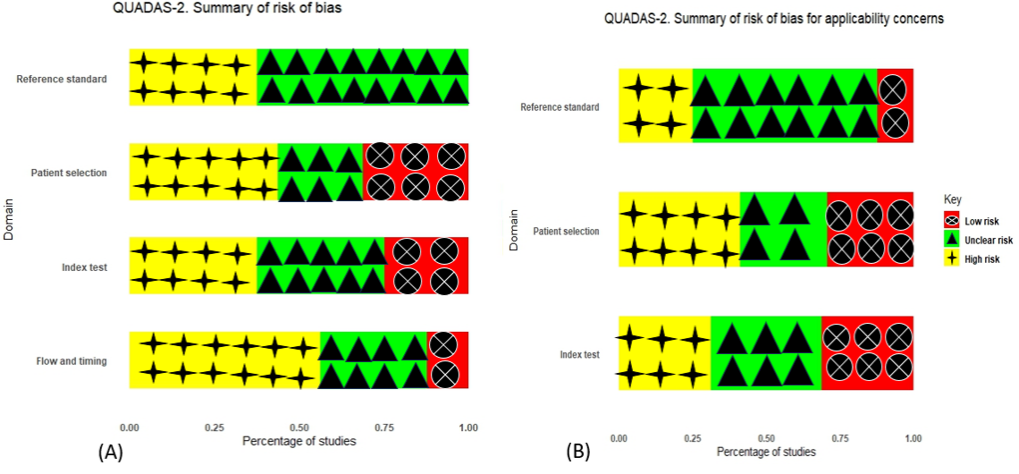
Summary of QUADAS-2 plots across the 16 included studies in the domain. (A) Risk of bias plot. (B) Risk of bias for applicability concerns. QUADAS-2: Quality Assessment of Diagnostic Accuracy Studies-2.

The quality assessment of the included studies was conducted using the Quality Assessment of Diagnostic Accuracy Studies-2 tool [21], a validated instrument designed to rigorously evaluate both the methodological integrity and reporting quality of diagnostic accuracy studies. This tool systematically appraises key domains, including patient selection, index test, reference standard, and flow and timing, thereby offering a structured framework for a comprehensive assessment of study quality. About 31% of the outcomes in the included studies lacked details on patient selection, leading to a high risk of bias, whereas the rest were considered low risk. In addition, 37% of the outcomes had unclear information on how the index test was performed, resulting in an unclear risk of bias for the test. Since the studies focused on AD, there was a mismatch between the review question and the index test. Furthermore, about 31% of the outcomes from all included studies did not clarify if the review question matched the targeted condition, raising concerns about the applicability of the index test. About 31% of the outcomes lacked clear information on the interval between the index test and reference standard, as well as on their execution, leading to unclear bias regarding flow and timing. The remaining studies were considered low risk for these factors. Figure 2 shows the overall study of the risk of bias evaluation.

### Statistical Analysis

The analysis combining data from multiple studies was conducted independently for sensitivity and specificity to consolidate the diagnostic accuracy findings of each network model. In this study, we used a random-effects model [22] to analyze the diagnostic performance of various ML models for medical diagnosis.

The primary outcomes are sensitivity, specificity, and the summary receiver operating characteristic curve, to provide a comprehensive assessment of diagnostic performance across studies. We computed point estimates and their corresponding 95% CIs for sensitivity and specificity to ensure consistency and comparability. Secondary outcomes, such as positive and negative likelihood ratios, were also explored. To assess statistical heterogeneity, the Cochran Q test and the *I*^2^ statistic were used. For the Q statistic, values ranging from 0% to 40% imply insignificant heterogeneity, 30% to 60% connote moderate heterogeneity, and 75% to 100% mean considerable heterogeneity. Publication bias was assessed using funnel plots, with significance set at *P*<.05 for 2-sided tests. Furthermore, we conducted subgroup analyses by stratifying studies based on the training approach, distinguishing between those using the ViT model alone and hybrid models. This meticulous approach allowed for a robust evaluation of the effectiveness and potential biases within the literature.

## Results

### General Overview of All Included Studies

**Figure 1:**
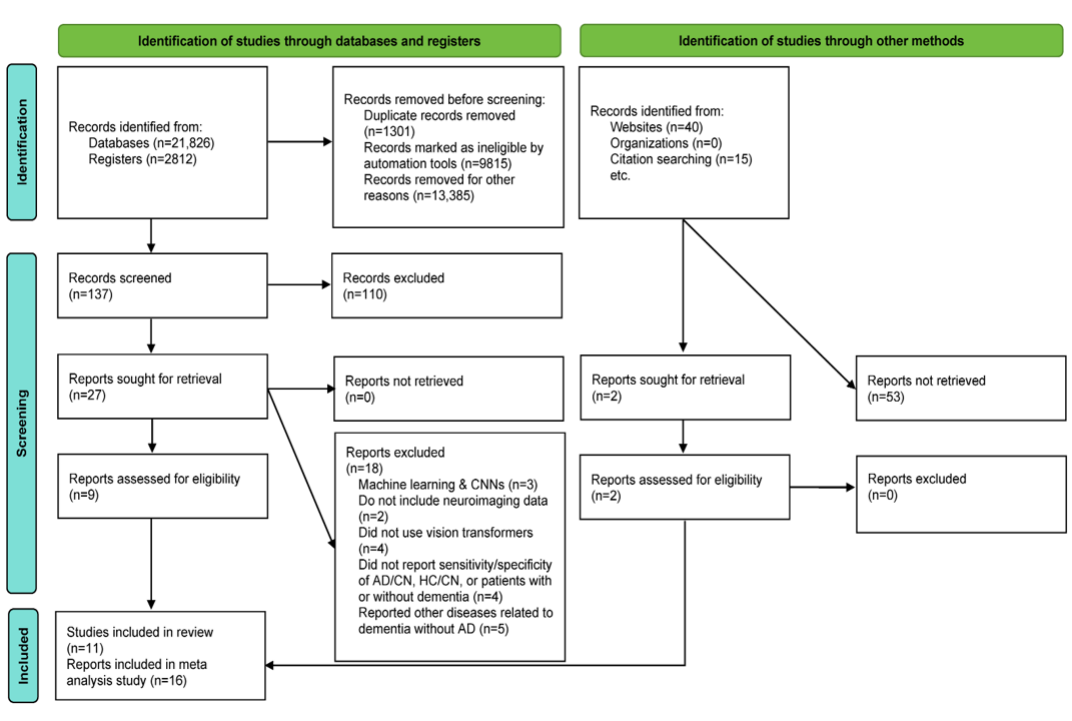
PRISMA flowchart for search strategy. Records were identified from CENTRAL, China National Knowledge Infrastructure, PubMed, Scopus, ScienceDirect, and Web of Science. AD: Alzheimer disease; CN: cognitively normal; CNN: convolutional neural network; HC: healthy controls; PRISMA: Preferred Reporting Items for Systematic Reviews and Meta-Analyses.

The initial search across databases yielded a total of 24,693 publications. After removing duplicates and screening the titles and abstracts, 11 papers [23-33] met the eligibility criteria, and 16 reports were included for qualitative synthesis and meta-analysis [23-33]. In addition, 4 of these papers [24,26,30,32] reported more than one study, resulting in a total of 16 outcomes that were used in the analysis as shown in Figure 1.

The ViT-based network architectures were classified into 2 categories. Among the reported studies, 7 of 16 outcomes (64%) used ViT models alone during the training and testing phases, whereas the remaining studies used hybrid models that integrated ViT with other DL architectures for training and testing. Various public data sets were used for training and evaluating the models, including the Alzheimer’s Disease Neuroimaging Initiative; Alzheimer’s Disease Neuroimaging Initiative Grand Opportunity Study; UK Biobank; Australian Imaging, Biomarkers, and Lifestyle Study; and others. The neuroimaging modalities used in these studies encompassed functional MRI, structural MRI, and PET, specifically fluorodeoxyglucose-PET.

The characteristics of the included studies are summarized in Table 1. The studies originated from China, representing 36% (4/11) of the total. The United States is the second most frequent at 27% (3/11). Lithuania and Korea are tied for third at 9% (1/11) each, whereas Colombia and Thailand each account for 9% (1/11). The most common objective across these studies was the diagnosis of AD versus NCs. Some studies used different datasets, and in such cases, we included each study separately. In addition, certain studies reported on multiple network architectures.

**Table 1:** Comprehensive summary of the characteristics of all the studies included in this systematic review.

Study	Country of study	Network architecture model (ViT^a^/hybrid)	Data (imaging method), MRI^b^/PET^c^	Training epochs/batch size	Type of task	Significance and the limitations of the study	Brief study summary
Alejandro et al 2023 [23]	Colombia	ViT	MRI	250/NA^d^	Classification	Significance: new image preprocessing techniques and spatial data augmentation. Limitations: small data set	This study uses deep learning to analyze MRI scans and detect AD^e^ with up to 89% accuracy in early stages.
Odusam et al 2023_1 [24]^f^; Odusam et al 2023_2 [24]^f^	Lithuania	ViT	MRI+PET	NA/1024	Detection	Significance: using transfer learning, which improves the performance of the proposed model; an image fusion technique has been proposed to fuse multimodal images for AD diagnosis. Limitations: limited data set (ADNI^g^ only used), the fusion parameters in the study were not optimized to their full potential.	Multimodal MRI+PET fusion for improved AD.
Zhu et al 2022 [25]	China	ViT	MRI	16/NA	Detection	Significance: integrates representation learning, feature distilling, and classifier modeling into a unified framework. Limitations: the fixed 5×3×5 size of the feature extraction patch is less suitable because brain disease-related structural changes vary in size, so a dynamic patch size would be more appropriate.	BraInf, a novel model, uses long-term connections in brain scans to detect AD with near-perfect accuracy.
Tang et al 2023_1^f^ [26]; Tang et al 2023_2 [26]^f^; Tang et al 2023_3^f^ [26]	China	Hybrid	MRI+PET	128/300	Detection or classification	Significance: in the study CsAGP^h^ a dual-branch vision transformer using cross-attention and graph pooling for multilevel feature interaction and representation was introduced to learn a shared feature representation by interacting with features at multiple levels. Limitations: the proposed CsAGP is slice-based and considers only axial view slices. Since 2D images cannot include all the information from a full brain scan. Also, this study has not conducted a time processing comparison.	A dual-branch vision transformer model using cross-attention and graph pooling for AD detection from multimodal images, achieving high classification accuracy rates through extensive experiments on the ADNI database.
Liu et al 2023 [27]	China	Hybrid	MRI	50/NA	Classification	Significance: a multimodal method used to handle missing data in various clinical settings. Limitations: reliance on a GPU^i^ may pose challenges in clinical environments where access to GPUs is limited.	This work introduces a cascaded multimodal mixing transformer (3MT) model for AD classification that effectively handles incomplete data, leveraging cross-attention and a novel modality dropout mechanism for robust and state-of-the-art performance on multimodal data sets.
Dhinagar et al 2023 [28]	United States	ViT	MRI	16/NA	Classification	Significance: variants of the ViT architecture used, 3D MRI images. Limitations: small data set used.	Application of ViTs on structural MRI scans for AD detection, achieving high classification accuracy.
Pan et al 2022 [29]	China	Hybrid	MRI	NA/NA	Classification	Significance: GAN^j^ and transformer architecture used in AD detection. Limitations: one dataset used.	A Cross-Modal Transformer GAN (CT-GAN) framework that effectively fuses structural and functional brain imaging data to enhance AD prediction was introduced.
Khatri et al 2024_1 [30]^f^; Khatri et al 2024_2 [30]^f^	Korea	ViT; hybrid	PET	32/NA	Classification	Significance: ViT architecture by pretraining the feature extractor using the self-distillation with no labels (DINO) and extreme learning machine (ELM) as classifier models was introduced. Limitation: training process is complex.	RMTnet, a deep learning model combining recurrent neural networks (RNNs) and transformers for AD diagnosis using FDG-PET, was introduced.
Khan et al 2024 [31]	Thailand	Hybrid	MRI+PET	100/32	Detection	Significance: a novel model of mixed-transformer with furthered U-Net architecture. Limitations: lack of model interpretability.	The model combines MRI and PET scans with mixed-transformer and semantic segmentation techniques, achieving superior performance metrics compared with existing models.
Aghdam et al 2024_1 [32]^f^; Aghdam et al 2024_2 [32]^f^	United States	ViT; hybrid	MRI	20/NA	Detection	Significance: diagnosis of AD on white matter of T1-weighted structural data. Limitations: small data sets.	PVTAD^k^, a new method for AD diagnosis that applies the Pyramid Vision Transformer to the white matter of T1-weighted structural MRI data, was introduced.
Huang et al 2023 [33]	United States	Hybrid	MRI	50/16	Classification	Significance: combining CNN^l^ and ViT. Limitations: low accuracy.	A self-supervised learning approach for dementia diagnosis from MRI scans was introduced, achieving state-of-the-art 92% accuracy.

^a^ViT: vision transformers.

^b^MRI: magnetic resonance imaging.

^c^PET: positron emission tomography.

^d^NA: not available.

^e^AD: Alzheimer disease.

^f^Studies that reported more than 1 outcome, for 3 outcomes (1,2,3) and 2 outcomes (1,2).

^g^ADNI: Alzheimer’s Disease Neuroimaging Initiative.

^h^CsAGP: cross-attention and graph pooling algorithm.

^i^GPU: graphic processing unit.

^j^GAN: generative adversarial network.

^k^PVTAD: pyramid vision transformer for Alzheimer disease.

^l^CNN: convolutional neural networks.

### The Overall Diagnostic Test Accuracy

**Figure 3:**
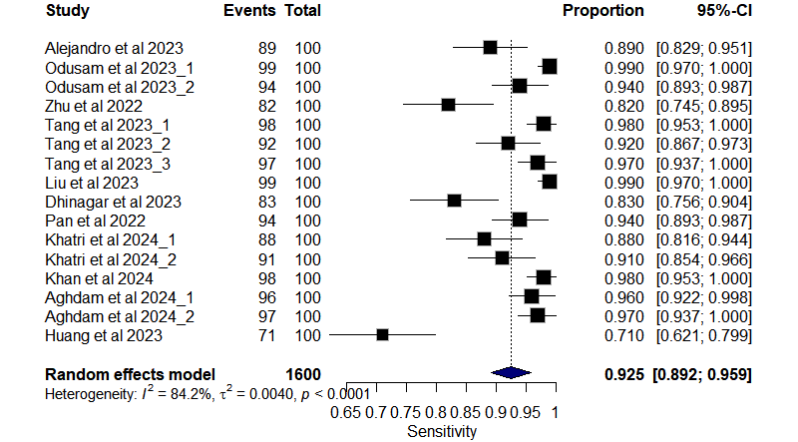
Forest plot of pooled sensitivity from 11 studies [23-33] with 16 outcomes using a meta-analysis of the proportion random-effect model.

**Figure 4:**
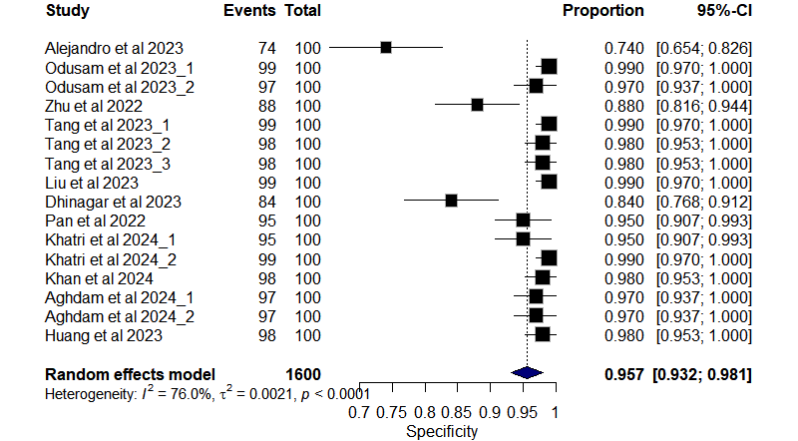
Forest plot of pooled specificity from 11 studies [23-33] with 16 outcomes using a meta-analysis of the proportion random-effect model.

The overall DTA for all included studies is presented in Figure 3; the pooled sensitivity of univariate analysis of 11 studies [23-33] with 16 outcomes was 0.925 (95% CI 0.892-0.959, *I*^2^=84.2% for 16 outcomes). The pooled specificity of univariate analysis was 0.957 (95% CI 0.932-0.981, *I*^2^=76% for 16 outcomes), as shown in Figure 4.

**Figure 5:**
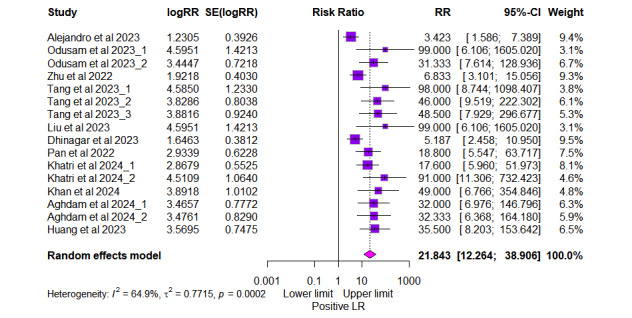
Forest plot of pooled positive likelihood ratio (LR) from 11 studies [23-33] with 16 outcomes. The risk ratio (RR) with 95% CI for each outcome is presented alongside the corresponding study weight.

**Figure 6:**
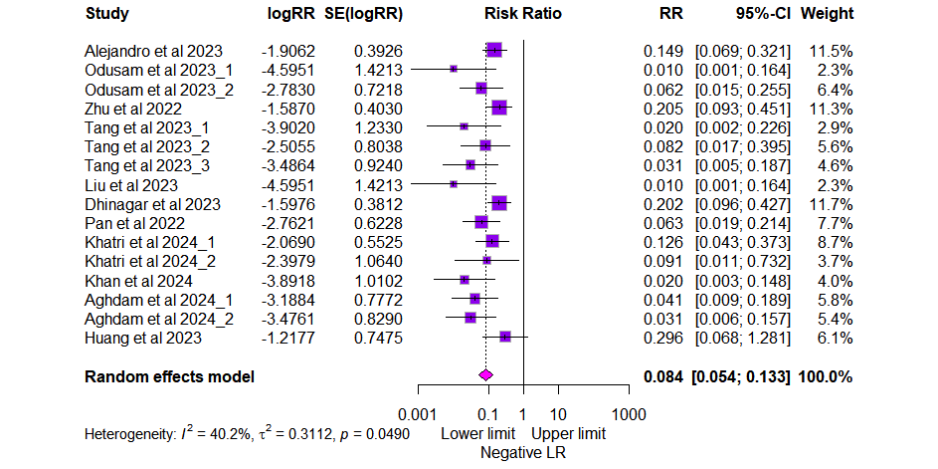
Forest plot of pooled negative likelihood ratio (LR) from 11 studies [23-33] with 16 outcomes. The risk ratio (RR) with 95% CI for each outcome is presented alongside the corresponding study weight.

**Figure 7:**
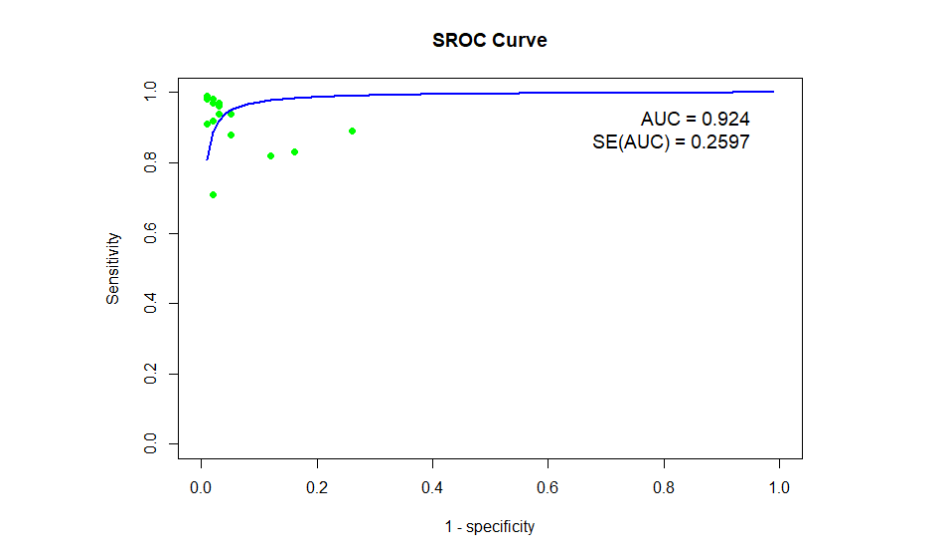
A plot of bivariate SROC for the diagnosis of AD using the ViT model. Green points represent individual studies included in the analysis, while the blue curve represents the fitted SROC curve based on the bivariate model. AD: Alzheimer disease; AUC: area under the curve; ViT: vision transformer; SROC: summary receiver operating characteristic curve; SE(AUC): standard error of the area under the curve.

As depicted in Figure 5, the positive likelihood ratio (LR+) showed a pooled value of 21.84 (95% CI 12.26-38.91, *I*^2^=64.9% for 16 outcomes). Similarly, the negative likelihood ratio (LR–) showed a pooled value of 0.084 (95% CI 0.054-0.133, *I*^2^=40.2% for 16 outcomes), as shown in Figure 6. The summary receiver operating characteristic curve of the bivariate model has an AUC of 0.924 (Figure 7), indicating excellent overall diagnostic performance.

### DTA for the Subgroup Analysis Based on Network Architecture

**Figure 8:**
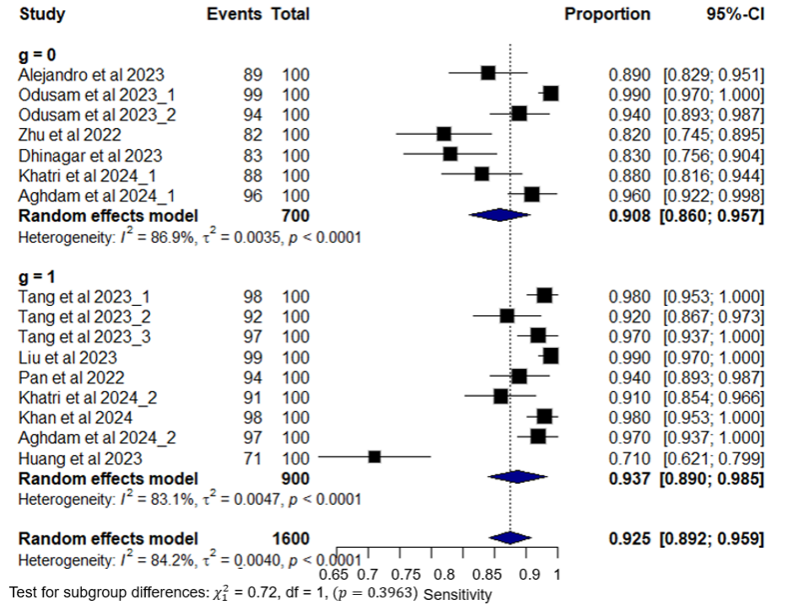
Subgroup analysis plot of pooled sensitivity based on the network architecture category. NB: Model 0 represents the ViT model alone, whereas Model 1 adapts the ViT model with other deep learning (DL) models.

Based on the network architecture, the model was divided into 2 models: ViT models and hybrid networks that incorporate the ViT model with other DL models. The ViT model alone has a sensitivity of 0.908 (95% CI 0.860-0.957, *I*^2^=86.9% for 7 of 16 outcomes), whereas the hybrid models have a sensitivity of 0.937 (95% CI 0.890-0.985, *I*^2^=83.1% for 9 of 16 outcomes). The pooled sensitivity values indicate that there is no significant difference between the 2 subgroups of the network model, with a *P* value of .40, as shown in Figure 8. The pooled specificity of the ViT model alone exhibits a pooled specificity of 0.912 (95% CI 0.849-0.976, *I*^2^=88.4% across 7 of 16 outcomes). In contrast, the hybrid model demonstrates superior pooled specificity at 0.984 (95% CI 0.975-0.992, *I*^2^=0% across 9 of 16 outcomes). These results reveal that there is a statistically significant difference in the specificity of the 2 categories of network models with a *P* value of .03 as shown in Figure S1 in Multimedia Appendix 3.

In the subgroup analysis comparing the ViT model and the hybrid model in our meta-analysis, the pooled DOR for the ViT model was 152.681 (95% CI 34.969-666.632, *I*^2^=87.4% for 7 of 16 outcomes), whereas for the hybrid model, it was notably higher at 880.203 (95% CI 337.53-2052.12, *I*^2^=50% for 9 of 16 outcomes). In addition, the *P* value for the subgroup difference was found to be .04, indicating a statistically significant difference between the 2 models. Given the substantially higher pooled DOR for the hybrid model and the significant subgroup difference, it shows that the hybrid model appears to be more effective in diagnosing AD based on our meta-analysis findings (Figure S2 in Multimedia Appendix 3).

### Assessment of Study Quality

Assessment of the review domains on risk of bias and applicability concerns was performed after reviewing studies. The review domains included patient selection, index test, reference standard, and flow and timing.

#### Patient Selection

There were 4 of 16 outcomes from 3 studies [28,30,33] (25%) that raised low concerns for patient selection bias and 5 of 16 outcomes from 2 studies [26,32] (31.25%) with high concerns, whereas 7 of 16 outcomes from 6 studies [23-25,27,29,31] (43.75%) were unclear, due to the studies not providing the detailed information about dataset used during training, testing, and validation.

#### Index Test

There were 6 of 16 outcomes from 4 studies [26,28,29,33] (38%) that raised low concerns, 4 of 16 outcomes from 3 studies [25,27,32] (25%) with high concerns, and 6 of 16 outcomes from 4 studies [23,24,30,31] (38%) that were unclear, primarily due to inadequate reporting of methodological details.

**Reference Standard**

There were 10 of 16 outcomes (62%) from 7 studies [25,26,28,29,31-33] with low concerns and 6 of 16 outcomes (38%) from 4 studies [23,24,27,30] that were unclear.

**Flow and Timing**

We conducted applicability concerns based on the interval between the index test and reference standard, whether all patients were included in the analysis, and whether the same data set used in studies received the same reference standard. The results showed 7 of 16 outcomes from 4 studies [28,30,31,33] (43.75%) raise low concerns for patient selection bias, 3 of 16 outcomes from 2 studies [26,32] (18.75%) raise high concerns, and 6 of 16 outcomes from 5 studies [23-25,27,29] (38%) were unclear; for the index test, 4 of 16 outcomes from 4 studies [26,28,29,33] (25%) raise low concerns, 5 of 16 outcomes from 4 studies [25,27,31,32] (31.25%) raise high concerns, and 5 of 16 outcomes from 3 studies [23,24,30] (31.25%) were unclear; for reference standard, 10 of 16 outcomes from 7 studies [25,26,28,29,31-33] (62%) raise low concerns, 1 of 16 outcomes from 1 study [30] (6%) raises high concerns, and 4 of 16 outcomes from 3 studies [23,24,27] (25%) were unclear, which can be found in Multimedia Appendix 4.

## Discussion

### Principal Findings

In the current systematic review and meta-analysis, the DTA of the vision transformer (ViT) in detecting AD was evaluated. The pooled DTA metrics across 11 studies revealed robust performance in detecting AD. The pooled sensitivity was 0.925 (95% CI 0.892-0.959), indicating the model’s ability to correctly identify individuals with AD, whereas the specificity was 0.957 (95% CI 0.932-0.981), demonstrating its capacity to accurately classify individuals without AD. In addition, the positive likelihood ratio was 21.84 (95% CI 12.26-38.91), showing the strength of a positive test result, whereas the negative likelihood ratio was 0.08 (95% CI 0.05-0.13), indicating the robustness of a negative test result. The area under the curve was 0.924, further confirming the model’s overall diagnostic accuracy. Comparatively, the study conducted by Odusami et al [7] also assessed diagnostic performance in AD detection using ML and DL models. Their meta-analysis reported pooled sensitivity and specificity estimates of 94.60% (95% CI 90.76-96.89) and 93.49% (95% CI 91.60-94.90), respectively, for differentiating AD from CN individuals. Notably, while our specificity estimate was slightly higher than that reported by Odusami et al [7], both studies demonstrate high accuracy in AD detection.

The comparison suggests that both approaches achieve strong diagnostic performance, with minimal variations in specificity estimates. These differences may originate from variations in sample characteristics, imaging protocols, or model architectures used across studies. Overall, the findings corroborate the effectiveness of ML and DL approaches in AD detection, providing valuable insights for clinical practice and future research directions.

Qu et al (2022) [9] conducted a meta-analysis on AD versus CN classification across 11 studies, revealing GAN-based DL with a pooled sensitivity of 0.88, specificity of 0.93, and an AUC of 0.96. In our study, using the ViT-based models for AD detection, we observed promising results with a pooled sensitivity of 0.940, specificity of 0.962, and an impressive AUC of 0.9874. Our findings suggest that ViT models offer high diagnostic accuracy in distinguishing patients with AD from healthy controls, potentially enabling early and accurate diagnosis. Notably, our results further indicate slightly superior sensitivity and specificity compared with GAN-based approaches, indicating the potential superiority of ViT models in AD detection.

Hu et al [34] conducted a meta-analysis assessing the diagnostic performance of MRI-based ML in AD detection. Their findings revealed a DOR of 43.34 (95% CI 26.89-69.84), suggesting strong discriminatory power, as the odds of a positive result were 43.34 times higher in patients with AD compared with those without AD. Comparatively, our analysis indicates that ViT-based models outperformed other types of DL models. Hu et al [30] reported an LR+ of 7.15 (95% CI 5.40-9.47) and an LR– of 0.17 (95% CI 0.12-0.22). These likelihood ratios imply that a positive result is associated with a higher likelihood of AD, whereas a negative result suggests a lower likelihood. In contrast, our estimated results for ViT models yielded a pooled LR+ of 26.02 and LR– of 0.07. This indicates that a positive ViT model result is 26.02 times more likely in individuals with AD compared with those without, whereas a negative result is more likely in patients without AD. Overall, the findings highlight the superior diagnostic accuracy of ViT models in AD detection compared with other types of DL models.

The meta-analysis revealed substantial heterogeneity across the studies due to differences in countries, ViT-based architectures, and data types. This heterogeneity could be a potential source of bias and highlights the need for more studies to obtain more consistent results in AD detection. The high sensitivity and specificity of the ViT-based models imply that ViT models can be a valuable tool in clinical scenarios to aid radiologists and neurologists in interpreting neuroimages for AD, potentially reducing the number of false negatives and false positives. The use of ViT, which captures direct relationships between image areas, may offer advantages in analyzing the complex network of the brain. Based on a search of academic literature databases, this is the first systematic review and meta-analysis that focuses on the diagnosis of AD using ViT-based models. Therefore, this review serves to highlight the potential of ViT and neuroimaging in enhancing the precision and productivity of AD diagnosis, providing a plethora of innovative solutions to address the challenges inherent in early detection and classification. However, this study faced some limitations. First, the pioneering nature of this systematic review presents a challenge for comparative analysis with other DL models. This is because, to the best of our knowledge, there have been no previous systematic and meta-analysis studies that focus specifically on ViT in the context of AD. The pioneering nature of this study means that there is a lack of existing literature to draw comparisons and establish a benchmark for the diagnostic accuracy of ViT-based models. The study acknowledges this issue and recognizes the need for further research to fill this gap. In the future, more studies are needed; specifically, there is a need to establish a more comprehensive understanding of ViTs diagnostic accuracy and their performance relative to other DL models in diagnosing AD.

Second, the reliance on public datasets for the included studies means that the diagnostic performance of ViT methods is based on data that may not fully represent the complexity and variability of real-world AD cases. The use of such datasets, although useful for initial research, does not account for the potential subtleties and specificities of patient data obtained from hospitals. This limitation is further compounded by the challenges associated with patient privacy and the complexities of preprocessing hospital data, which can hinder the validation of ViT methods in a real-world context. The study emphasizes the need for future research to address these challenges and validate the findings on more representative and diverse datasets. Researchers should also conduct further evaluations of ViTs using hospital data to better align with real-world applications [35].

Third, the limited number of studies available has restricted the research to binary classification tasks, which differentiate patients with AD from CN individuals. This focus does not capture the full spectrum of AD's progression, including the various stages and the transition from MCI to late MCI and stable MCI [15]. The study acknowledges the need for a broader application of ViT-based DL methods to detect and track the various stages of AD, which is crucial for a more comprehensive understanding of the disease and its progression. In addition, there is a notable gap in studies that use multimodal data to predict the progression of AD.

Furthermore, the study highlights a significant gap in the current models' ability to provide expert-level interpretations [36]. The absence of a reference standard in the form of expert diagnosis makes it difficult to conclusively determine the superiority of ViT-based models in real-world health care settings. This limitation underscores the importance of future research efforts that should focus on enhancing the transparency and explainability of ViT-based models. In addition, there is a need to explore the integration of these models with expert opinions to improve the accuracy of AD detection and diagnosis. By doing so, the research can move toward a more collaborative approach that combines the strengths of both artificial intelligence and human expertise.

Third, most ViTs require the use of GPUs for training, which may not be readily available in many hospitals. Therefore, there is a need to develop smaller models that can be trained in more affordable and accessible environments.

Finally, the findings of this study have important implications for clinical practice. ViT models show high diagnostic accuracy in AD detection, offering the potential for early diagnosis and personalized treatment. However, several barriers may hinder their implementation in routine clinical settings. These include the need for large, well-labeled datasets, the risk of overfitting, and the challenges of integrating ViT models into existing clinical workflows. In addition, the computational resources required for training, such as GPUs, may not be readily available in many hospitals. Addressing these challenges is essential to realize the full potential of ViTs in clinical applications.

### Conclusion

The systematic review and meta-analysis highlight the strong diagnostic capability of ViT-based models in detecting AD from MRI and PET brain images. Although there are limitations, such as the lack of comparative analysis with other DL models, ViT models demonstrate high sensitivity and specificity. ViTs have the potential to significantly enhance image analysis in AD diagnosis by improving feature extraction, enabling early detection of subtle brain changes, supporting personalized diagnoses, and facilitating the integration of additional data for more accurate diagnoses. However, the effective application of ViT models comes with challenges, including the need for large datasets, the risk of overfitting, and the integration of these models into clinical workflows. To fully harness their potential in clinical settings, continued research is essential, particularly to improve model transparency and incorporate expert opinions. This study highlights the promising role of ViTs in advancing early AD detection and classification, paving the way for innovative solutions in neuroimaging analysis.

## Appendix

Multimedia Appendix 1 Search terms used in each database.

Multimedia Appendix 2 PRISMA checklist.

Multimedia Appendix 3 Supplementary figures for subgroup analysis of diagnostic test accuracy (DTA) vision transformers based on network architecture.

Multimedia Appendix 4 Supplementary table for Risk of Bias assessment.

## Abbreviations

AD: Alzheimer disease
CENTRAL: Cochrane Central Register of Controlled Trials
CNN: convolutional neural networks
DL: deep learning
DOR: diagnostic odds ratio
DTA: diagnostic test accuracy
LR+: positive likelihood ratio
LR–: negative likelihood ratio
ML: machine learning
MRI: magnetic resonance imaging
NC: normal control
PET: positron emission tomography
ViTs: vision transformers
