# Supplementary material for: Detection of Alzheimer Disease in Neuroimages Using Vision Transformers: Systematic Review and Meta-Analysis
Source: J Med Internet Res. 2025 Feb 5;27:e62647. doi: 10.2196/62647 (PMC11840381; doi:10.2196/62647)
Supplement: Multimedia Appendix 1 [file jmir_v27i1e62647_app1.docx]

**Search Terms used in each database**

1. **PubMed Search Terms using MeSH**

("Alzheimer Disease"[MeSH] OR "Dementia"[MeSH])

AND ("Transformers, Artificial Intelligence"[MeSH] OR "Deep Learning"[MeSH])

AND ("Neuroimaging"[MeSH] OR "Brain"[MeSH]) AND ("Classification"[MeSH] OR "Diagnosis"[MeSH])

**Search link for PubMed Database**

Search link here: <https://pubmed.ncbi.nlm.nih.gov/?term=%28%22Alzheimer+Disease%22%5BMeSH%5D+OR+%22Dementia%22%5BMeSH%5D%29+AND+%28%22Transformers%2C+Artificial+Intelligence%22%5BMeSH%5D+OR+%22Deep+Learning%22%5BMeSH%5D%29+AND+%28%22Neuroimaging%22%5BMeSH%5D+OR+%22Brain%22%5BMeSH%5D%29+AND+%28%22Classification%22%5BMeSH%5D+OR+%22Diagnosis%22%5BMeSH%5D%29&filter=dates.2020%2F1%2F1-2024%2F2%2F28>

1. **Refined Search Terms for Web of Science Database**

(((ALL= (Alzheimer Disease*)) AND ALL= (Vision Transformer*)) OR ALL=(ViT*)) AND ALL=(Classification*)

**Search link for Web of Science Database**

<https://webofscience.clarivate.cn/wos/woscc/summary/d44ac402-89a5-4d7e-8bf1-5834bd19fc01-011e9adcd2/relevance/1>

1. **Refined Search Terms for CNKI Database**

**Search link** **for CNKI Database**

<https://scholar-cnki-net-s.webvpn.usst.edu.cn/home/search?ad=1>

**Scopus,Cochrane Central Register Of Controlled Trials (Central),Science Direct**

1. **Refined Search Terms for Scopus Database**

("Alzheimer Disease" OR "Dementia") AND ("Vision Transformer" ) AND ("Neuroimaging" OR "Brain Imaging" ) AND ("Detection" OR "Diagnosis")

**Refined Search link** **for Scopus Database**

<https://bnu1.sjuku.top/https/77726476706e69737468656265737421e7e056d2343367406b1bc7af9758/results/results.uri?sort=plf> f&src=s&sid=e918a9d4efee357a0984278538cf808c&sot=a&sdt=cl&sl=71&s=%22Alzheimer%26apos%3Bs+Disease%22+AND+%28%22Vision+Transformer%22%29+AND+%28%22Detection%22%29&origin=resultslist&editSaveSearch=&txGid=a3db990bcb82eb80fab49c30fa692fa1&sessionSearchId=e918a9d4efee357a0984278538cf808c&limit=10&yearFrom=2021&yearTo=2024

1. **Refined Search Terms for CENTRAL Database**

("Alzheimer Disease" OR "Dementia") AND ("Vision Transformer" ) AND ("Neuroimaging" OR "Brain Imaging" ) AND ("Detection" OR "Diagnosis")

**Refined Search link** **for CENTRAL**

<https://www.cochranelibrary.com/advanced-search?cookiesEnabled>

**Refined Search Terms for Science Direct**

[Keywords ((Alzheimer Disease OR AD) AND (vision transformer OR ViT) AND (Neuroimaging OR Brain imaging) AND (classification OR detection OR diagnosis )) Year(2020-2024) - Search | ScienceDirect.com](https://www.sciencedirect.com/search?qs=(Alzheimer%20%20Disease%20OR%20AD)%20AND%20(vision%20transformer%20OR%20ViT)%20AND%20(Neuroimaging%20OR%20Brain%20imaging)%20AND%20(classification%20OR%20detection%20OR%20diagnosis%20)&date=2020-2024)
