# Supplementary material for: Detection of Alzheimer Disease in Neuroimages Using Vision Transformers: Systematic Review and Meta-Analysis
Source: J Med Internet Res. 2025 Feb 5;27:e62647. doi: 10.2196/62647 (PMC11840381; doi:10.2196/62647)
Supplement: Multimedia Appendix 3 [file jmir_v27i1e62647_app3.docx]

**Supplementary figures illustrating the subgroup analysis of diagnostic test accuracy (DTA) for the ViT model by network architecture.**


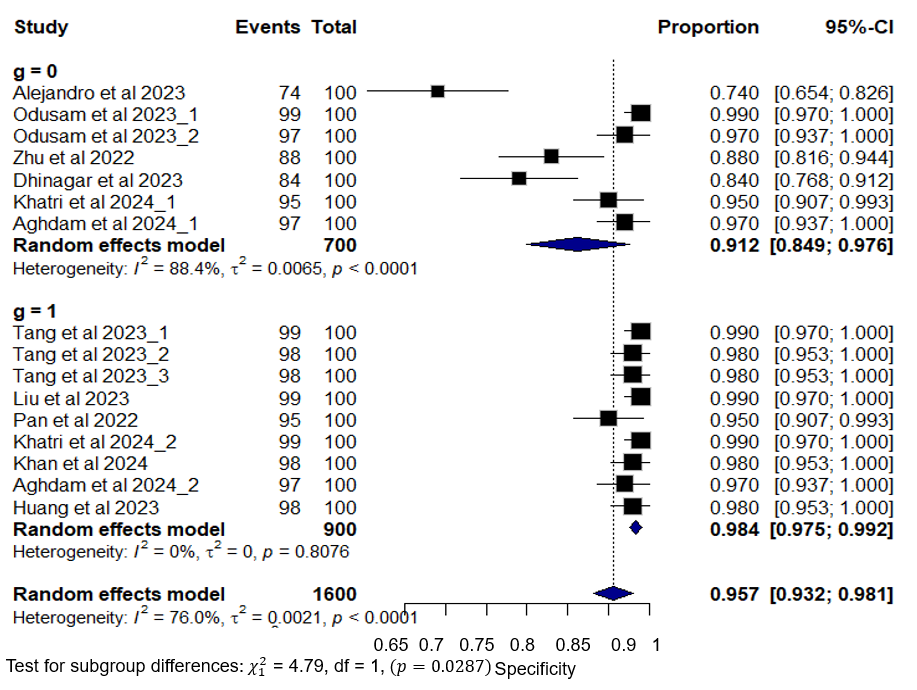


**Figure 9.** Subgroup analysis plot of pooled specificity based on the network architecture category. NB: Model 0 represents the ViT model alone, whereas Model 1 adapts the ViT model with other deep learning (DL) models.


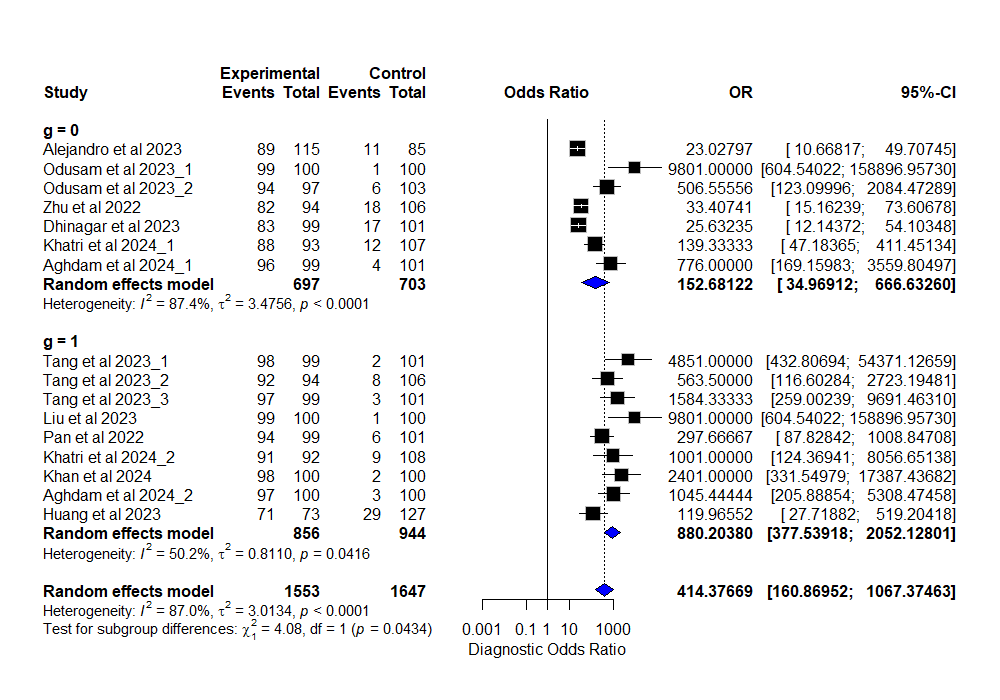


**Figure 10.** Subgroup analysis of pooled diagnostic odds ratio (DOR) comparing ViT and hybrid networks. NB: Model 0 represents the ViT model alone, whereas Model 1 is the hybrid that adapts the ViT model with other deep learning (DL) models.
