# Supplementary material for: Detection of Alzheimer Disease in Neuroimages Using Vision Transformers: Systematic Review and Meta-Analysis
Source: J Med Internet Res. 2025 Feb 5;27:e62647. doi: 10.2196/62647 (PMC11840381; doi:10.2196/62647)
Supplement: Multimedia Appendix 4 [file jmir_v27i1e62647_app4.docx]

Table S1: Supplementary Table for Risk of Bias Assessment

|  | **Study type** | **Risk of Bias Applicability Concerns** | | | | | | |
| --- | --- | --- | --- | --- | --- | --- | --- | --- |
|  |  | **Patient Selection** | **Index Tests** | **Reference Standard** | **Flow and Timing** | **Patient Selection** | **Index Tests** | **Reference Standard** |
| 1. | Alejandro et al. 2023 [23] | Unclear | Unclear | Unclear | High | Unclear | Unclear | Unclear |
| 2. | Odusam et al. 2023_1 [24] | Unclear | Unclear | Unclear | Unclear | Unclear | Unclear | Unclear |
| 3. | Odusam et al. 2023_2 [24] | Unclear | Unclear | Unclear | Unclear | Unclear | Unclear | Unclear |
| 4. | Zhu et al. 2022 [25] | Unclear | High | Low | Unclear | Unclear | High | Low |
| 5. | Tang et al. 2023_1 [26] | High | Low | Low | Low | High | Low | Low |
| 6. | Tang et al. 2023_2 [26] | High | Low | Low | Low | High | Low | Low |
| 7. | Tang et al. 2023_3 [26] | High | Low | Low | Low | High | Low | Low |
| 8 | Liu et al. 2023 [27] | Unclear | High | Unclear | Unclear | Unclear | High | Unclear |
| 9. | Dhinagar et al. 2023 [28] | Low | Low | Low | Unclear | Low | Low | Low |
| 10. | Pan et al. 2022 [29] | Unclear | Low | Low | High | Unclear | Low | Low |
| 11. | Khatri et al.2024_1 [30] | Low | Unclear | Unclear | Low | Low | Unclear | High |
| 12. | Khatri et al.2024_2 [30] | Low | Unclear | Unclear | Low | Low | Unclear | High |
| 13. | Khan et al.2024 [31] | Unclear | Unclear | Low | Unclear | Low | High | Low |
| 14. | Aghdam et al.2024_1 [32] | High | High | Low | Unclear | High | High | Low |
| 15. | Aghdam et al.2024_2 [32] | High | High | Low | Unclear | High | High | Low |
| 16. | Huang et al. 2023 [33] | Low | Low | Low | Unclear | Low | Low | Low |
